# Pooling arrangements in health financing systems: a proposed classification

**DOI:** 10.1186/s12939-019-1088-x

**Published:** 2019-12-21

**Authors:** Inke Mathauer, Priyanka Saksena, Joe Kutzin

**Affiliations:** 1 Department of Health Systems Governance and Financing, World Health Organization, Avenue Appia, 1211 Geneva, Switzerland; 2 World Health Organization, Tunis, Tunisia

**Keywords:** Health financing, Pooling, Fragmentation, Equity

## Abstract

**Objectives:**

The function of pooling and the ways that countries organize this is critical for countries’ progress towards universal health coverage, but its potential as a policy instrument has not received much attention. We provide a simple classification of country pooling arrangements and discuss the specific ways that fragmentation manifests in each and the typical challenges with respect to universal health coverage objectives associated. This can help countries assess their pooling setup and contribute to identifying policy options to address fragmentation or mitigate its consequences.

**Methods:**

The paper is based on a review of published and grey literature in PubMed, Google and Google Scholar and our information gathered from our professional work in countries on health financing policies. We examined the nature and structure of pooling in more than 100 countries across all income groups to develop the classification.

**Findings:**

We propose eight broad types of pooling arrangements: (1.) a single pool; (2.) territorially distinct pools; (3.) territorially overlapping pools in terms of service and population coverage; (4.) different pools for different socio-economic groups with population segmentation; (5.) different pools for different population groups, with explicit coverage for all; (6.) multiple competing pools with risk adjustment across the pools; and in combination with types (1.)-(6.), (7.) fragmented systems with voluntary health insurance, duplicating publicly financed coverage; and (8.) complementary or supplementary voluntary health insurance. However, we recognize that any classification is a simplification of reality and does not substitute for a country-specific analysis of pooling arrangements.

**Conclusion:**

Pooling arrangements set the potential for redistributive health spending. The extent to which the potential redistributive and efficiency gains established by a particular pooling arrangement are realized in practice depends on its interaction and alignment with the other health financing functions of revenue raising and purchasing, including the links between pools and the service benefits and populations they cover.

## Introduction

Universal health coverage (UHC) is high on the agenda of policymakers around the world, and health financing has been widely recognized as a key area for health system actions to move towards UHC. Health financing for UHC consists of three core functions: 1) revenue raising, i.e. the mobilization of resources for the health sector; 2) pooling, i.e. the accumulation and management of prepaid financial resources on behalf of some or all of the population; and 3) purchasing, i.e. the allocation of pooled funds to health service providers [1]. Whereas revenue raising, e.g., [2–5] and purchasing [6–10] have been receiving strong academic and policy interest over the years, pooling arrangements and their potential to contribute to progress towards UHC have received much less attention. Only a few publications [1, 11–16] provide conceptual insights into the structure of and mechanisms for pooling arrangements. Yet, the function of pooling and the different ways that countries organize this is critical for countries’ progress towards UHC. Risk pooling is the spreading of the financial risk associated with the need to use and pay for health services, rather than to be fully borne by the individual who falls ill [11].The objectives of this paper are to raise the profile of pooling as a health financing policy instrument and to provide a simple classification of country pooling arrangements through which we discuss the challenges typically associated with how fragmentation manifests in each setting. In turn, this can help countries assess their pooling arrangements and contribute to identifying policy options to address fragmentation or mitigate its consequences. However, as with any classification, it is a simplification of reality, and the aim is not merely to categorize a country in one type or another. While we believe that the classifications are useful, they are not a substitute for the detailed work that is needed in any one specific country to fully understand its pooling arrangements, their links to other health financing and system functions and their implications for policy. Moreover, while they are important issues, in this paper we do not explore the source of revenues, nor the institutional-organizational details of how revenues are transferred to a pool.

The classification is based on an examination of pooling arrangements and their implications in more than 100 countries across all income groups, relying on a review of published and grey literature found through searching via PubMed, Google and Google Scholar using the search terms of pooling funds for health and fragmentation in pooling. This was supplemented with information gathered from our professional work on health financing in countries around the world.

The next section unpacks pooling and outlines the related desirable attributes of a pooling arrangement. This is followed by an outline of the key institutional design aspects of pooling arrangements and how these can create fragmentation. Based on this, we identify and present broad types of pooling arrangements and related fragmentation issues and discuss implications and challenges. For illustration we provide various country examples. A conclusion and lessons are presented at the end.

## Pooling as an objective and a policy instrument, and what makes for good pooling arrangements

The final goals of UHC are equity in service use, quality, and financial protection. Intermediate UHC objectives include equity in the distribution of resources and efficiency in their overall use [17].

Improved equity in service use and financial protection involve expanding risk pooling, and as such *pooling is a policy objective* in itself. Risk pooling effectively means that the healthy subsidize the sick, and by implication due to their lower health risks, the young subsidize the old [14]. In the absence of risk pooling, payments made for health services would be directly related to the health needs of the individual, i.e. “sicker” individuals would have to pay more because they would need more health services [18]. This is inconsistent with the objective of financial protection and equity of access to services in relation to need. Therefore, maximizing the potential to redistribute from lower-need to higher-need individuals by de-linking contributions (of whatever form, such as taxes or insurance premiums) from their health risk is the central objective for pooling. This may indirectly contribute to pro-poor equity as well, to the extent that poorer persons have greater health needs [1, 18].

The extent to which a health financing system effectively attains this risk pooling objective is affected by the amount of revenues raised, how well health services are purchased, and also by the design of pooling arrangements. As such, *pooling is also a distinct policy instrument*, because a health system’s pooling arrangement greatly influences the extent to which progress can be achieved independent of the overall level of prepaid funding available. Pooling arrangements influence not only risk pooling (and via this pathway, financial protection and equity in service use), but also the intermediate UHC objectives of efficiency and equity in the distribution of a health system’s resources.

*Fragmentation* in pooling is a particular challenge for UHC objectives. Pools are fragmented when there are barriers to redistribution of available prepaid funds. Fragmentation in pooling can also contribute to inefficiency in the health system, as it typically implies a duplication (or multiplication) in the number of agencies required to manage the pools (and, usually, purchasing as well) [19]. This is due to two related reasons. First, there are higher administrative costs of having multiple pooling/purchasing agencies rather than one, which can raise system-wide costs. Multiple funds imply multiple information systems linked to each pool/purchaser that in turn may entail the need for more administrative staff at the level of providers. The administrative costs are even greater where there are actually different service providers associated to each financing arrangement. This duplication of functional responsibilities can be a major driver of inefficiency when seen from the perspective of the entire system rather than within each scheme [15, 20]. Second, fragmentation can weaken the potential gains from using purchasing as an instrument to influence provider behavior in countries where multiple purchasers use different payment methods and rates to pay the same providers in an uncoordinated way. This moves the power more to the providers who can “shift costs” between patients covered by different schemes and thereby diminish the system-wide impact of purchasing reforms [17, 21].

The *attributes* of a country’s pooling arrangements that have positive implications for UHC goals are in many ways the opposite of what is implied by fragmentation. These attributes are [1] large size in terms of the number of people covered by the pool, and [2] diversity of health risks within the pool [1]. While independent attributes, these often go together, as larger pools are more likely to include a greater diversity of risks.

## Institutional design aspects of pooling arrangements

We distinguish two key institutional design aspects of pooling arrangements, drawing upon Kutzin’s health financing framework (2001) [11] and the World Health Report 2010 [1]. These are 1) the nature of pooling and 2) the structure of pooling. Table 1 outlines the respective features under each.

**Table 1:** Key institutional design aspects and their features

Nature of pooling	Structure of pooling
Voluntary versus compulsory or automatic coverage	Single versus multiple pools within multiple pools*:* - territorially distinct versus territorially overlapping pools in terms of service and population coverage - competing versus non-competing - population segmentation versus no population segmentation

Source: compiled by authors

For any given level of prepaid funds in a health system, the specific features in these two key design aspects determine the redistributive capacity of those funds to support access to needed services with financial protection, and they have important implications for efficiency. It is the various combinations of the different features in the structure and in the nature of pooling that drove our classification of pooling arrangements described in the next section. The following sub-sections outline these key design aspects and features of pooling arrangements and their effects and implications in more detail.

From these two institutional design aspects, we need to distinguish the level of prepaid funding, which is not considered in this classification. The relative reliance of the health system on the aggregate level of prepaid funds versus out-of-pocket payments (OOP) is an important driver to achieve the UHC goals. However, the overall level of prepaid funds arises from how a health system raises revenues, and not how it organizes pooling arrangements. (Of course, fragmented pool structures will yield more dependence on OOP expenditure and thus decrease the share of prepaid funds in overall health spending). Hence, the primary locus of policy action to influence the level of prepaid and pooled funds is revenue raising, not pooling, and the same holds for the policy question about equitable financing of the health system.

### Nature of pooling: compulsory versus voluntary

Pools can be based on compulsory, automatic or voluntary participation. Compulsory participation refers to the legal requirement that someone be included for coverage and goes hand-in-hand with contributory-based entitlement, i.e. there must be a specific contribution made by or on behalf of the covered person. The “on behalf” may come from public budgets for specific groups of individuals whose participation is fully or partially subsidized, or it may come from traditional insurance contributions that cover individuals beyond the contributor (e.g. family members). Automatic participation is typically based on legal or constitutional obligations, and the basis for entitlement is non-contributory, deriving from citizenship, residence or other factors such as poverty status, etc. As such, automatic entitlement is typically solely funded from general budget revenues. Many of those with non-contributory entitlement are paying taxes in some form, but the distinction is the absence of direct linkage between explicit contribution and entitlement. Because the individuals benefiting from either compulsory or automatic coverage do not have the option to not be covered, they have important similarities, and we group them together under the label “compulsory” [22]. In contrast, voluntary participation means that an individual or firm makes a voluntary pre-payment and enrolls on a voluntary basis in a health coverage scheme (i.e. voluntary health insurance). It is voluntary because there is no legal obligation to join a scheme, and thus the person or their employer can choose not to be part of a pool for coverage [22]. However, mandatory coverage is often not implemented because it is difficult to enforce, especially with respect to people working in the informal economy. The result is that even where it is legally mandatory for the entire population, it is de facto voluntary coverage.

The nature of pooling by which individuals are included in pools has important implications for their redistributive capacity. When coverage is compulsory or automatic for all population groups, the pool(s) have a more diverse mix of health risks. People who have higher risks are just as covered as people who have lower risks. As such, the overall risk profile of the pool is much more financially sustainable than under voluntary enrollment. Conversely, schemes that have voluntary membership, i.e. voluntary contributions from beneficiaries, are prone to adverse selection: people with higher risks are more likely to enroll than people with lower health risks. As a result of inadequate diversity of healthier and sicker people, the costs of care for a pool based on voluntary coverage are in principle higher than for the average in the population. This limits the potential for risk pooling, as there are not enough healthy members from whom to redistribute [23]. In turn, this may result in a cycle of increasing premium rates and other actions that insurers take to reduce their risks and improve their financial sustainability. Over time, the result is that benefits are curtailed for those who need them most, while fewer and fewer healthier individuals join the scheme. This is the so-called “death spiral” of voluntary health insurance [24].

### Structure of pooling: single versus multiple pools

In any country, prepaid health revenues may be held, i.e. pooled, in one or several pools. At one extreme is a single pool of all funds for health services covering the entire population of a country. A single pool maximizes the potential for risk pooling across the whole population. Taken quite literally, perhaps no country has only one single pool. Even in countries with highly centralized pooling, there are usually several pools of funds that are used to pay for some health services, for example occupational health programs, supply-side funding for other government services such as those delivered through vertical programs or voluntary health insurance [1].

But the key concern is that the existence of multiple pools implies fragmentation. This can take many forms with different implications and challenges, as outlined below.

#### Competing versus non-competing pools

It is possible to have competition across pools, i.e. agencies that manage pools (typically insurance schemes) compete for members. Alternatively, in a non-competitive arrangement, people could be assigned to specific pools, with enrollment being based on explicit criteria, so that the different pools cannot compete for beneficiaries [11].

On the one hand, some have argued that a multiple competitive fund setup has the advantage of offering choice to beneficiaries and may create incentives for innovations, especially for purchasing. However, evidence for efficiency improvement with increased market competition among purchasers is weak [25]. On the other hand, competition among insurance pools creates an incentive for pool managers to ‘cream skim’, i.e. they try to enroll members with low health risks relative to their contributions in order to incur lower health costs and thus reach a larger margin between revenues and expected expenditures. The investments that competing insurers make to try and select preferred risks (or avoid high health risks) are inefficient from a social welfare perspective [11, 26], because the resources devoted to risk selection do not contribute to progress towards UHC, and in fact may detract from it. Risk selection negatively affects the redistributive capacity, as healthier and wealthier individuals and their contributions often end up in a different pool than poorer and sicker members with (usually) lower contributions. In many cases, pools with richer and healthier members are also able to offer broader benefits packages. Conversely, pools with higher health risks are more likely to restrict benefits (if this is legally allowed), face financial difficulties or else run deficits. Risk selection practices can be addressed with risk adjustment mechanisms (which we discuss further below in the next section).

#### Population segmentation versus no population segmentation

A multiple pool setup can be based on population segmentation, i.e. there are different funds for different population groups, with the affiliation being based on socio-economic or (socio-) demographic criteria. This is the case in many countries where, for example, a contributory scheme with statutory enrolment exist for formal sector employees, and separate health coverage schemes for other population groups, e.g. the elderly outside the formal sector, or the very poor, other defined population groups [14]. Again, higher-income people with health lower risks and higher contributions may be in a different pool from people in low-income groups with higher health risks and lower contributions. Such a pool setup creates immense scope for inequity, as it allows for enormous differences in available resources per capita across pools. Conversely, there is no population segmentation when coverage and participation in a pool is independent of people’s socio-economic or (socio-)demographic criteria.

#### Territorially distinct versus territorially overlapping pools in terms of service and population coverage

Pools may be organized as territorially distinct. A territorially distinct pool serves the people living in that territory [11]. Pools are thus not divided along population groups. Instead, they usually follow a country’s territorial structure, i.e. a sub-national pool per state, province or district. When each level of government in a decentralized setting pools for a distinct level of health services, then it is organized in a territorially distinct way. For example, district governments only pool for ambulatory care and district level hospitals, provinces for provincial hospitals, and the national government for high-level tertiary services. But where territorially distinct pools are too small in terms of the number of people, their risk profile can be financially precarious and there could be efficiency and capacity concerns. Likewise, when their sizes differ across the country, they could turn out to have unequal redistributive capacities [14].

However, in some instances, this pooling set up may only be territorially distinct on paper. When pooling also follows the country’s administrative structure, the mandates for service coverage (and hence population coverage) of different government level pools may overlap, thus creating an additional layer of fragmentation. For example, the pool from which the national capital city funds its “city hospitals”, and the pool from which the central government funds national tertiary facilities are not territorially distinct, particularly when – as is often the case – the national tertiary hospital is also an important provider of more basic services for the local population. This overlap turns into duplication of service coverage particularly in big cities, with the main policy consequence being large inefficiencies in the form of excess provider capacity [15].

## Types of pooling arrangements and fragmentation issues

**Fig. 1:**
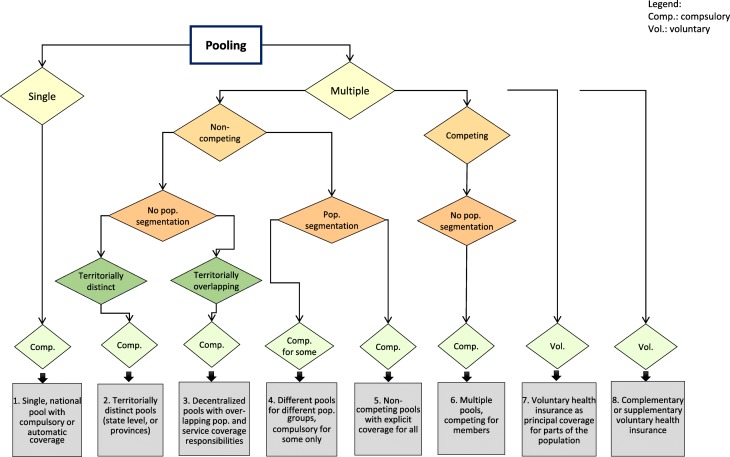
Classification of pooling arrangements

To develop the classification, we combined the different features in the structure and the nature of pooling and then examined the nature and structure of pooling in more than 100 countries across all income groups. Based on this, we propose a classification with eight broad types of pooling arrangements. These tend to reflect particular challenges due to the nature and consequences of fragmentation in each. The classification is presented in Fig. 1 below. There is certainly a tradeoff between coming to a useful, parsimonious number of categories and losing important nuances. An additional layer of complexity is that in many countries several forms of fragmentation exist. Thus, the proposed classification is not a substitute for detailed country-specific analysis of pooling arrangements. Rather it is a first attempt at a classification, which could encourage further useful work from others.

It is important to mention that supply side financing, where the “health budget” flows in a vertically integrated way to service providers, constitutes a pool, and in many cases is often the largest pool in low- and middle-income countries. It serves multiple purposes, e.g. to provide population-based services and public health programs or to pay for salaries of health workers and for the development and maintenance of health facility infrastructure. This health budget pool is included in the pooling arrangements outlined below and is also often characterized by fragmentation.

### (1.) Unified single pool with compulsory or automatic coverage

There are some countries that rely predominantly on a single national pool funded from general government revenues. Such a pool provides compulsory or automatic coverage for the entire population, usually for a defined package of services. There are two forms of institutional setup found for this pooling arrangement.

Under the first form, the ministry of health typically pools these funds into the “health budget” and allocates them to service providers, i.e. there is no explicit purchaser-provider split. All people have in principle access to the same benefits. In practice, only a few countries have this pooling arrangement alongside a low share of out-of-pocket expenditure (OOP) (< 20% of total health spending). Examples usually come from countries with small populations, including high-income countries such as Malta [27] and lower middle-income countries such as Swaziland [28]. Cuba, with a much larger population, also has this setup, as does Sri Lanka, where financial protection performance is relatively good despite a high share of OOP [29].

Under the second form of institutional setup, countries have established a single national fund that is managed by a separate pooling and purchasing agency, usually with a purchaser-provider split. The agency is typically labelled as a national health insurance fund and constituted as an autonomous public entity. This entity pools public funding, i.e. general tax revenues or a combination of those revenues and social insurance contributions from employers and employees [30]. This type of pooling arrangement is also usually found in countries with relatively small populations, such as Costa Rica, Estonia, Lithuania, Moldova and Mongolia [31–34]. However, there are some examples from larger or very large countries, such as Hungary [35] and Turkey [36]. Indonesia is also undertaking efforts to shift towards a single national health insurance pool, but there is still a significant part of the population that is not yet enrolled in the pool. Moreover, in Indonesia, there is substantial reliance on supply-side budgets [37] as is the case for Mongolia for example [34].

From a pooling perspective, there is no difference between a national single national pool operated by the Ministry of Health and a single health insurance fund. Maximum redistributive capacity from prepaid funds is achievable in these settings. Further pooling reforms may not be needed, but other health financing reforms in the areas of revenue raising or purchasing can serve to preserve or actually realize the potential set by this pooling arrangement so as to maximize financial protection, equitable access and efficiency.

### (2.) Territorially distinct pools with compulsory or automatic coverage

Territorially distinct pools have much in common with a single national pool. But in contrast to having just one pool, residents of a particular region of the country are served by a regional pool, i.e. there is one fund for the population in that one territory. Here the pooling function lies with a sub-national entity, such as a state, province, or district (if managed by a level of public administration) or another entity, such as a health insurance fund, with defined responsibility for the entire population of that territory [14]. Systems relying on territorially distinct pools are usually a product of a wider political context of federalism or devolution. The resources allocated to these different pools may come from a mix of centrally and sub-nationally raised revenues, with allocations often based on a consistent formula applied across the country.

But a system with territorially distinct pools can suffer from fragmentation, if and when their population size or the territory are too small to ensure redistributive capacity, or when sub-national territories have very different levels of average per capita expenditure on health. Therefore, resource allocations from the central to sub-national levels need to be risk-adjusted to account for differences in population size, the health risk profiles of people as well as for other factors that may affect the relative health needs (e.g. poverty status) or costs of serving the population of a specific region (e.g. population density). Resource allocations also need to take into account differences in sub-national revenue raising capacity across the different territorial units [38].

Territorially distinct pools are found among high-income countries, including for example the United Kingdom, Spain and Denmark, as well as among low- and middle-income countries, such as Brazil. Sometimes, these arrangements include a purchaser-provider split. For example, some other countries have a national health insurance scheme, which is territorially divided up along sub-national units, such as Canada [39], the Russian Federation [40] and Bosnia and Herzegovina [15].

### (3.) Territorially overlapping pools in terms of service and population coverage

Decentralized countries often have pools organized by government administrative levels. Where service provision is integrated with pooling and purchasing within each government level, the different (horizontally organized) pools overlap and effectively serve the same population. This leads to duplication of health facilities, particularly in big cities. This was, and in some places remains, one of the main drivers of large inefficiencies in the health systems of the ex-USSR countries [15].

### (4.) Population segmentation through different pools for different socio-economic groups

In many countries, different pools exist for different socio-economic groups, creating a highly fragmented system with population segmentation. Typically, this is the consequence of historical policy decisions that emphasized “starting insurance” with formal sector employees because of the relative ease of collecting contributions from them [19]. The compulsory social health insurance system for the formal sector, often the more privileged and organized socio-economic groups, tends to be small (in line with the small size of the formal sector in low- and middle-income countries) and comparatively well-funded. In contrast, the public budget through the Ministry of Health offers theoretically free health services for the rest of the population. But services are typically grossly underfunded and often unavailable, thus resulting in implicit benefits [1]. Fragmentation is further aggravated, as a small part of the better-off population is often enrolled in commercial voluntary health insurance, whilst a small share of people in the informal sector may enroll in voluntary community-based health insurance schemes [41, 42]. Finally, there may be specific coverage schemes for defined population groups, such as the poor [30].

Such pooling setups create explicitly unequal financing arrangements and the population segmentation is often further linked with separate purchasing and service provision arrangements. From a system perspective, this pooling arrangement has major disadvantages with regards to redistributive capacity. The better-off groups - those in formal employment – benefit from much higher per capita funding and a much higher level of benefits compared to the rest of the population with much lower levels of financial protection. Indeed, these arrangements put in place for health financing further exacerbated existing inequalities in these countries rather than compensating for them.

While the issue of segmentation first emerged in Latin America [43], it is not limited to that region. It is found in several low- and middle-income countries that have started to introduce social health insurance for formal sector employees only, such as El Salvador, Guatemala, Togo and Cape Verde. In some cases, this is limited to civil servants only.

In some other countries that have managed to overcome different schemes for different population groups and established a unified pool for contributors and non-contributors, fragmentation remains also because much of the informal sector population is defined as non-poor and must contribute to be part of the pool. As a consequence of this de facto voluntary arrangement, countries such as Ghana, the Philippines and Vietnam still experience inequities between the insured and uninsured population [34, 44].

### (5.) Different pools for different population groups, with explicit coverage for all

Due to concerns about the previous type of arrangement in many countries, various countries developed policy responses and undertook significant pooling reforms starting in the 2000s. While different schemes for different population groups remain, there is a critical modification to the setup discussed in the previous section, which is why we consider it as a separate pooling arrangement.

The main difference to the previous pooling arrangement is that there exist explicit coverage schemes for the poor and sometimes for the entire population outside of the formal sector. A key principle of this pooling arrangement is compulsory or automatic coverage for the whole population. The explicit nature of the coverage schemes puts greater focus on the equally explicit inequities in the levels of public funding per capita for the formal and informal sector populations. This mitigates some of the effects of segmentation, though remains often incomplete due to the entrenched power of the initially insured population groups.

Thailand is a prominent example for this pooling arrangement. In the early 1990s, Thailand had a scheme for civil servants and another scheme for private sector employees. It also had schemes for the low-income population and the elderly and a subsidized voluntary insurance program for the rest of the population. These latter three were replaced by a new health coverage scheme that was introduced in 2002, called the Universal Coverage Scheme (UCS), as a response to growing concerns about the huge differences in level of funding per capita across the schemes and the remaining coverage gap due to the failure of the voluntary insurance to reach much of the informal sector. The UCS pooled together all of those revenues plus increased budget allocations. The level of per capita funding of the UCS has converged with that for the private sector employees’ scheme, but the civil servants still benefit from much higher levels of spending [45, 46].

Mexico’s Seguro Popular also shifted to this principle of automatic coverage of all people who are not part of an insurance scheme for formal sector employees [47, 48]. Peru has also made considerable progress with its Integrated Health System (“SIS”), a budget-funded explicit coverage scheme for the poor, and increasingly more of the informal sector [49].

Common to these low- and middle-income country examples is that they did not manage to merge all coverage schemes into one pool due to the resistance of the formal sector employees for a unified national scheme. These countries had therefore decided to create an explicit coverage program for people outside the formal sector, whilst trying to gradually increase the level of funding to narrow the gap in per capita expenditure across the different schemes. Although this pooling arrangement does not fully overcome fragmentation and population segmentation, it substantially reduces it.

### (6.) Multiple competing pools with compulsory coverage and risk adjustment across the pools

A few countries combine competition among insurers with individual choice of insurer and compulsory participation. This is commonly referred to as a competitive social health insurance arrangement. Each of the insurance schemes thus constitutes a separate pooling agency. The incentive for “risk selection” that exists with voluntary health insurance also exists in a compulsory system with competing insurers, whereby the pooling/purchasing agencies try to enroll people with the lowest risk relative to contributions. This has an adverse impact on equity in resources across pools. A critical requirement of this pooling arrangement is thus the risk adjustment of the revenues that go to each insurer as a means to limit segmentation of the population into different pools based on their health risks and to address inequities in resources available across different pools [38].

Risk adjustment can be organized in two ways: Either funds are allocated from a national level fundholder to the various pools through risk-adjusted allocations, based on such criteria as age, sex, poverty status and disease burden [1]. Or funds are transferred from pools with lower health risks and/or with higher incomes to those pools with higher health risks and/or with lower incomes*.*

As such, this type of pooling arrangement, if and when it has an effective risk adjustment mechanism that deters risk selection efforts, can act as a virtual single pool (due to the flows between the pools). It has important similarities with the (2.) type of pooling arrangement, namely territorially distinct pools. In particular, the aim in both is to match the level of per capita funding of each pool with the relative health risk of the population affiliated to each pool.

Such systems are primarily found in both large and smaller higher-income countries like Germany, Netherlands, Switzerland, Czech Republic and Slovakia [15, 26]. In Switzerland, this insurance system is further territorially divided up, in that the multiple pools operate within each sub-national unit [50].

### (7.) Fragmented system: voluntary health insurance for a part of the population, duplicating publicly financed coverage schemes

Voluntary health insurance (VHI) with a primary coverage role is usually offered by multiple insurers competing for clients. When people have access to publicly financed coverage schemes, this VHI is duplicating. Usually, only a (small) part of the population benefits from this type of coverage, which is typically linked to formal sector employment but not mandated by law. Higher income persons are usually more likely to have this form of VHI [51].

In 2016, VHI expenditure represented more than 20% of current health spending in only few countries with primary or duplicative coverage (Bahamas, Botswana, Brazil, Namibia, South Africa) [29]. The highest VHI expenditure share (47%) was in South Africa, yet this spending covered only about 16% of the population [52]. Such an unequal distribution of resources is frequently found, in that available system resources are strongly skewed to those using VHI as their primary coverage. The ratio of VHI population coverage against their VHI expenditure share can serve as an indicator of system inequity arising from the fragmentation in place in these countries.

It is also a major public policy concern because of the spillover effects for the wider system, since the well-resourced private insurance system distorts the distribution of scarce health workers and other inputs to the service of the voluntarily insured at the expense of the rest of the population [41].

### (8.) Voluntary health insurance with either a complementary or supplementary role

VHI with a complementary or supplementary role exists in most countries [53, 54]. As the name suggests, it exists in addition to and along the other main pooling arrangements, as outlined above. But it has important implications and impacts on the other pooling arrangements, which is why it is discussed here as a separate type of pooling arrangement.

Complementary insurance *for user charges* complements coverage of the public system by covering all or part of the residual costs (e.g. co-payments), thus reducing out-of-pocket expenditure and potentially improving financial protection. Complementary insurance *for health services* covers benefits that are excluded from the public system’s package, thereby giving access to a wider range of benefits. Supplementary insurance, on the other hand, provides enhanced access, such as a higher level of inpatient amenities or greater user choice of providers compared to the coverage in the public system [51, 55].

From a system point of view, there are benefits to this arrangement because these forms of VHI can fill explicit gaps in publicly funded coverage. There are also some concerns, however. Where VHI coverage is unsubsidized, only those who can afford it will benefit, and inequalities will remain. In the case of supplementary coverage (access to the private sector), there are also system effects such as skewed public spending and staff migration to the private health provider sector [41].

However, in most countries with complementary or supplementary VHI, VHI expenditure is below 10% of current health expenditure [29], and when a large part of the population has this form of VHI coverage, spillover effects are less severe [22, 53]. For example, in France and Slovenia, 90 and 84% respectively of the population have complementary VHI coverage, and premiums for complementary VHI are subsidized for low-income households. This makes it affordable to them and addresses the inequity concerns that come along with complementary health insurance [53]. Moreover, in France, there is a shift towards compulsory complementary coverage, which employers have to buy for their employees since 2016 (with exceptions for various employee groups) [56].

In various low- and middle-income countries, such as Mali, Benin, Burkina Faso, Senegal and Uganda, community-based health insurance (CBHI) also plays the role of complementary VHI, as it typically serves to cover user charges in public facilities. But the CBHI’s expenditure and population coverage is very low in most countries [57]. Due to voluntary participation, small pool size and little or no subsidization of poor and vulnerable groups, CBHI can play only a very limited role in progressing towards UHC. Some countries, such as Rwanda and Ghana, have transformed their earlier CBHI model, which no longer falls under VHI. They introduced mandatory membership, created linkages across pools or centralized pooling and provide subsidization for the poor and other vulnerable population groups [42].

## Conclusion and policy lessons

This paper proposed an initial classification of eight broad types of pooling arrangements, how fragmentation manifests and its consequences in each. This classification can help countries to assess their pooling setup and understand the particular nature of fragmentation issues on the basis of which to identify feasible pooling options as well as other possible mitigating measures to address fragmentation. Among the eight types of pooling arrangements, types (3.) to (5.) and (7.) are deemed to be particularly problematic forms of fragmentation, because they strongly constrain redistributive capacity. They also contribute to system-wide inefficiencies arising from the duplication of responsibilities for managing different pools (with purchasing often linked to that).

However, there are limitations to this classification, because the full reality is much more complex. Multiple forms of fragmentation co-exist, and dimensions other than pooling also result in fragmentation. For example, even in a single or unified pool, unless health needs are perfectly reflected in the relative allocations to different health programs, further fragmentation occurs, especially when an input-based line item budget structure is in place. Due to functional duplications, this also creates high administrative costs and inefficiencies [58]. Fragmentation also occurs in the few countries (Germany, Netherlands, Chile) that allow certain population groups (e.g., the self-employed or individuals above an income threshold) to opt out from the public system and to buy mandatory private insurance [59–61].

Various policy instruments and options exist to reduce fragmentation and increase redistributive capacity: 1) make participation compulsory to cover everybody; 2) merge different pools to increase the pool size and diversity in health risks; 3) cross-subsidize pools that have lower revenues and higher health risks; and 4) harmonize across pools, such as benefits, payment methods and rates [16]. As changes in the pooling arrangements are about redistribution of funds, this is ultimately also very political, and it is hence important to understand the feasibility and manage the political economy of pooling reforms. However, relevant responses to improving pooling depend on the specific nature and the broader context of the country. The classification, such as the one we are proposing is simply meant to facilitate the reflecting around a response.

Finally, it is important to keep in mind that while pooling reforms are needed to enhance redistributive capacity, realizing the gains set by the potential of a pooling arrangement requires more than pooling. Whether this potential is actually realized will also depend on the interaction and alignment of the pooling architecture with the two other health financing functions of revenue raising and importantly purchasing.
